# Healthcare providers’ perspectives of providing primary healthcare services to persons with physical disabilities in rural Ghana

**DOI:** 10.1017/S1463423619000495

**Published:** 2019-07-01

**Authors:** Ebenezer Dassah, Heather M. Aldersey, Mary Ann McColl, Colleen Davison

**Affiliations:** 1 School of Rehabilitation Therapy, Queen’s University, Kingston, ON K7L 3N6, Canada; 2 Department of Public Health Sciences, Queen’s University, Kingston, ON K7L 3N6, Canada

**Keywords:** access to healthcare, Ghana, persons with physical disabilities, primary healthcare, rural health

## Abstract

**Background:**

A growing body of evidence has shown that persons with physical disabilities experience substantial barriers in accessing primary healthcare (PHC) services in rural areas. Negative attitudes from healthcare providers and inaccessible healthcare facilities and equipment are common experiences that negatively affect access to quality healthcare for persons with physical disabilities. However, there is limited research that explores this issue from the perspectives of healthcare providers. This qualitative study explores the perspectives of healthcare providers in delivering PHC services to persons with physical disabilities in rural Ghana. Understanding healthcare providers’ perspectives could help leverage previous findings from clients’ experiences to more fully inform the development of specific and actionable research and interventions to improve healthcare delivery for disabled people.

**Methods:**

We conducted in-depth interviews with 15 healthcare providers and used thematic analysis to analyze the data.

**Results:**

Participants reported their perspectives in three major themes: challenges in providing healthcare (eg, limited availability of drugs and medical equipment, limited healthcare providers, financial constraints, and inaccessible facilities and equipment); strategies in navigating the challenges (eg, improvising techniques and employing professional values, referring clients, and providing financial assistance to clients); and positive experience in providing healthcare (eg, feeling rewarded and appreciated).

**Conclusion:**

The findings reinforce the need to consider the availability of rehabilitation professionals and services to address the specific healthcare needs of disabled people at the PHC level in Ghana. The findings also point to a need for further research on the perspectives of healthcare policymakers about how to navigate the systemic barriers encountered by providers in providing care to persons with physical disabilities in rural Ghana in particular, and other similar rural areas.

## Introduction

The Convention on the Rights of Persons with Disabilities (UNCRPD) describes disabled people as those who experience long-term physical, mental, intellectual, or sensory impairments, which in interaction with various barriers may hinder their full and effective participation in society on an equal basis with others (United Nations, 2006). Disabled people constitute approximately 1 billion of the global population, with 80% living in low- and middle-income countries (LMICs) (World Health Organization, 2011). They are often regarded as the poorest and most marginalized in many communities (World Health Organization, 2011). Disabled people also often have limited access to healthcare as compared to their counterparts without disabilities (Badu *et al*., 2015). Given this, global policy documents including the Sustainable Development Goals and the UNCRPD have recognized the need for supporting disabled people’s access to quality healthcare. Ensuring equitable healthcare access for this population is also a top priority for healthcare systems in many LMICs (Dassah *et al*., 2018a; Tomlinson *et al*., 2009), including those in sub-Saharan Africa.

Ghana is one of the emerging countries of sub-Saharan Africa promoting and supporting access to healthcare for disabled people through the formulation of disability and health policies. For example, Ghana signed and ratified the UNCRPD, and also enacted the Persons with Disabilities Act in 2006 to protect the rights of disabled people (Government of Ghana, 2006). The Act has many provisions that aim to improve healthcare access for disabled people. For instance, it guarantees disabled people’s right to access the same quality and standard of healthcare as provided to other persons. Furthermore, the Act emphasizes free medical treatment and rehabilitation services. Aside from access to healthcare, disabled people’s right to access public spaces and services through the design of disability-friendly facilities are enshrined in this Act (Government of Ghana, 2006). Additionally, Ghana has been at the forefront in sub-Saharan Africa in ensuring universal access to healthcare for all its citizens. For instance, the government of Ghana introduced a national health insurance scheme in 2003, with the aim of increasing and improving access to quality and affordable healthcare for all residents regardless of socioeconomic status (Agyepong and Adjei, 2008). Premiums are charged to clients and are renewable on a yearly basis under the scheme. However, the scheme grants exemptions from these premiums for specific groups including elderly persons, children, social security contributors and pensioners, pregnant women, indigents (ie, extremely poor), and recently, those with mental health disorders. Payment of the annual premium fees’ exceptions are provided for disabled people classified as indigents within criteria set out by the government of Ghana (Badu *et al*., 2015; Dassah *et al*., 2018b).

Despite these policies, a growing body of evidence has shown that disabled people continue to experience barriers in accessing healthcare in Ghana (Badu *et al*., 2015, 2016a, 2016b; Ganle *et al*., 2016; Dassah *et al*., 2018b). In particular, a recent study indicated that persons with physical disabilities in rural Ghana encounter barriers that include negative attitudes from healthcare providers (ie, stigmatization and discrimination) and inaccessible healthcare facilities and equipment (Dassah *et al*., 2018b). Dassah *et al*. (2018b) exclusively sought the perspectives of persons with physical disabilities and revealed that these clients’ recollections of encounters with healthcare providers often cast the providers in a negative light. It is critical for new research to understand the perspectives of healthcare providers in order to have a holistic understanding of access for clients with physical disabilities especially in rural Ghana (Ganle *et al*., 2016; Dassah *et al*., 2018b). The objective of this study is to explore the perspectives of healthcare providers in providing care to persons with physical disabilities in rural Ghana. This study is timely in that healthcare providers are key stakeholders in improving healthcare access and quality for clients with physical disabilities. Hence, they can provide unique perspectives on how to translate policies into clinical practice, an area in which actors in national healthcare systems often struggle to make improvements (White, 2015). Further, healthcare providers’ perspectives will leverage previous findings from clients’ experiences (Dassah *et al*., 2018b) and develop specific intervention priorities to improve healthcare delivery for disabled people. In particular, healthcare providers’ perspectives will contribute to policy makers’ understanding of the barriers and facilitators in providing care to disabled people. Such understanding might shape decisions on the problems that need to be addressed to improve access to healthcare services for disabled people.

### Healthcare systems and care provision in rural Ghana

Healthcare delivery in Ghana is provided by both public and private sectors. The public or government-run healthcare sector provides two-thirds of all health services in Ghana (Institute for Health Metrics and Evaluation, 2015). The public health system in Ghana is organized in five tiers – Community-Based Health Planning and Services (CHPS) compounds at the community level, health centers at the sub-district level, district hospital at the district level, regional hospital at the regional level and specialized/teaching hospitals at the national level.

Primary healthcare (PHC) services are mostly provided at the community, sub-district and district levels in rural areas. At the community level, the CHPS compounds constitute the most basic level of healthcare services provision and function more to ensure quality and equitable PHC services for those living in rural and hard-to-reach communities. Under the CHPS compounds, healthcare providers deliver basic services to clients in their communities and homes. At the sub-district level, the health centers deliver both preventive and curative services. These centers especially provide minor surgical services and refer severe health cases to the next level of care. The district hospital constitutes the apex of healthcare delivery in rural areas. The facilities provide comprehensive care including curative care, preventive care, outpatient and inpatient services, and health promotion. The facilities in these three levels are mostly staffed with healthcare providers such as medical doctors, medical assistants, nurses, pharmacists, laboratory technicians, and auxiliary nurses (Agbenyo *et al*., 2017) – hereafter, we refer to these individuals as ‘providers’.

### Theoretical framework

The perspectives of providers are important in understanding access to healthcare. The concept of access to healthcare is complex and thus difficult to define and measure (Levesque *et al*., 2013). Andersen (1995) conceptualized access as both potential access (resources that enable clients to seek care) and realized access (clients’ actual use of care services). The use of service is determined by the interactions between the characteristics of the population and that of the healthcare system (Andersen, 1995). Thus, access to healthcare is achieved if health resources (supply) fit or interact with the individual healthcare need (demand) (Frenk, 1992). These demand and supply interactions have further been described by researchers as the ‘fit’ between the provider and the user in the following dimensions; accessibility, availability, affordability, accommodation, and acceptability (Penchansky and Thomas, 1981). More recent reviews have either modified or identified additional dimensions to operationalize healthcare access (Levesque *et al*., 2013; Russell *et al*., 2013; Saurman, 2016). For instance, Russell *et al*. (2013) suggested that access is a complex and multidimensional construct that includes the ‘fit’ between the client and the provider across seven dimensions – availability, geography, affordability, accommodation, timeliness, acceptability, and awareness. Access also encompasses the ability to identify and use health services in times of need (Levesque *et al*., 2013; Russell *et al*., 2013). In this study, we will conceptualize access based on Russell *et al*.’s (2013) framework because it focuses on rural perspectives of access to PHC.

## Methods

### Study design and setting

We used a descriptive qualitative approach to explore the perspectives of providers in delivering care to persons with physical disabilities in rural Ghana. A qualitative descriptive approach is well-suited for researching topics where practical answers are sought with relevance to healthcare practitioners and policymakers (Sandelowski, 2000). We obtained approvals from Queen’s University Health Sciences and Affiliated Teaching Hospitals Research Ethics Board and the Ghana Health Services.

The study was conducted in the Upper West Region of Ghana. Detailed information about the study setting is explained elsewhere (see Dassah et al., 2018b). In brief, the region has only 2.8% of Ghana’s population with 3.7% disabled people, and approximately 83.7% of the population is rural, thus making it the most predominant rural region in Ghana (Ghana Statistical Service, 2012).

### Participants and recruitment

We recruited 15 providers for the study (see Table 1). We believe that with this sample size, we achieved adequate ‘information power’ (when adequate sample size is defined in terms of study aim, sample specificity, use of established theory, quality of dialog, and analysis strategy) (Malterud *et al*., 2016). We used purposive sampling to recruit the participants based on the following inclusion criteria: (a) provision of health services to persons with physical disabilities in their health facilities – that is, persons with impairments that impact their physical functioning; (b) ability to communicate in English and/or Dagaare (dominant local language in study areas); and (c) willingness to participate. To achieve diverse perspectives, we sampled the participants based on age, sex, specialization, district, type of facility working, and number of years working in the profession (Table 1). We identified and recruited participants through the Upper West Regional Administration of Ghana Health Services. The Regional Administration wrote an introductory letter about the study to the study districts, thus granting permission to conduct the study. The first author visited the heads of health facilities who then introduced and/or provided the contact details of their staff to the first author. The first author contacted potential participants and informed them about the purpose of the study, risks and benefits from participation, prospective uses of collected information, and confidentiality of personal information. The first author interviewed participants who expressed interest and met the inclusion criteria at their convenient time and place. We provided participants with refreshments as a token of appreciation for their time and information shared.

**Table 1.** Characteristics of participants

Characteristic	Total, N
**Age (Years)**	
20–30	5
31–40	5
41–50	3
51–60	2
**Sex**	
Male	7
Female	8
**Specialization**	
General nurses	6
Medical assistants	4
Community health nurses	3
Medical doctor	1
Auxiliary nurse	1
**District**	
Daffiama/Bussie/Issa	3
Jirapa	4
Nadowli/Kaleo	5
Wa West	3
**Type of facility**	
Health centers	8
CHPS compounds	4
District hospital	3
**Years in profession**	
0–10	6
11–20	4
21–30	3
31–40	2

### Data collection, analysis and rigor

The first author conducted in-depth interviews (12 in English and 3 in Dagaare) in health facilities and at a time convenient to the participants. The interviews were digitally recorded with the participants’ consent and lasted between 45 and 60 min. The interview guide focused on participants’ roles, training in disability issues, and experiences in providing care to persons with physical disabilities. The first author simultaneously translated and transcribed the Dagaare interviews in English. Detailed information about the transcription and translation process is explained elsewhere (See Dassah *et al*., 2018b). In summary, the translated interview transcripts were crossed checked with two independent researchers fluent in both English and Dagaare. Similarly, the interviews in English were also transcribed verbatim by the first author and checked with the same independent researchers.

The data were then imported into NVivo™12 software and the first author conducted thematic analysis using the following major steps: (a) initial familiarization with the data, including repeatedly reading the interview transcripts while noting down key messages; (b) systematic generation of initial codes, including the creation of a code book; (c) tentative organization of codes into themes and sub-themes; and (d) iterative process of reviewing, restructuring, and refining of themes and sub-themes (Braun and Clarke, 2006). The first author collaborated with a second coder to code a sample of the transcripts to identify the themes. All the authors also discussed and reached consensus on the final themes.

## Results

Three major themes emerged from the analysis, and they include: (1) challenges in providing healthcare; (2) strategies in navigating the challenges; and (3) positive experiences in providing healthcare. Supportive quotations from the participants to demonstrate these themes and their related subthemes are summarized in Table 2 and described in the subsequent sections.

**Table 2.** Overview of themes and subthemes with supporting quotations

Themes	Subthemes	Quotations by participants
**Theme 1:** Challenges in providing healthcare	1. Limited supplies of drugs and medical equipment	*One day a client in a tricycle came to me with leakage of urine. I was thinking of giving him a catheter to manage the problem, but we don’t have some over here*.
		*We can’t get antibiotics for our clients with disabilities in here because there is a directive from the top…. Because of that clients who need them have to go to health centers or drugstores*.
	2. Limited healthcare providers	*One day a client with disability came to me with muscle spasm but I was unable to provide treatment to her because I do not have the necessary training to provide such service and also we don’t have any specialist at this level to address the needs of this client*.
		*For now, we are only two trained nurses that struggle to serve this community and beyond. And so on market days we usually have high workload. This sometimes negatively affects* *quality healthcare delivery to clients with disabilities*.
	3. Financial constraints	*Clients with disabilities who come here without health insurance always complained that they are not working and so don’t have money to cover medical expenses and other non-medical things like folder and consultation fees*.
		*Clients with physical disabilities often postpone visits to healthcare facilities because they don’t have money for transport. They also fear to walk or propel their tricycles far because of the fear of developing more complications*.
	4. Inaccessible facilities and equipment	*We have weighing scales here, but they are not disability friendly and so when those with severe physical disabilities cannot stand on them we don’t use them when they come for care*.
		*This facility is not that accessible for those with tricycles because we don’t have ramps at the entrances. The waiting room is also a small space for a tricycle to move freely*.
**Theme 2:** Strategies used in navigating the challenges	1. Improvising techniques and self-sacrifices	*For those clients in tricycles when they can’t get into the facility we (providers) usually try to lift the person in. But if we can’t do that we try to put a piece of wood between the gutters for the person to propel the tricycle into the building*.
		*Some clients with disabilities rely on their family members to come here. Sometimes they have to wait for their family members to return from work or school and if they (ie, family members) come back late, they could also come at the time I’m about to close. When this happens, I have to forgo domestic issues and rather attend to the person’s needs…. This usually happens on market days*.
	2. Referring clients	*Because we don’t have rehabilitation specialists over here, and so what I usually do is just to refer them to heath facilities that have these specialists*.
		*We don’t have antibiotics here and as a result I just give them (ie, clients with physical disabilities) referral note to health centers*.
	3. Providing financial assistance to clients	*I always feel that when clients with physical disabilities come here without money, it is my duty to financially assist them when I can’t get them all the needed care*.
		*I try to give them (ie, clients with physical disabilities) care and then use my own money to pay for those services because most them can’t afford for the services*.
**Theme 3:** Positive experiences in providing services	1. Feeling rewarded and appreciated in rural practice	*I feel happy and proud when I treat clients with disabilities. These clients always feel like neglected in the community and when they come for care they feel happy. Then that I realized how rewarding it is to stay in this community and provide services to them*.
		*When you provide care to these clients with physical disabilities and you meet them, they are always excited and thank you for the care. And this really makes me happy*.
		*I was surprised to see a young man who came and gave me some tubers of yams (farm produce) just to express how appreciative he is for the service I provided to his family member with a physical disability*.

### Theme 1: Challenges in providing healthcare

#### Limited availability of drugs and medical equipment

A notable challenge highlighted by all the participants was the frequent lack of drugs and medical equipment in health facilities. Providers especially noted that delay in payment of services by the national health insurance scheme hindered the supply of drugs and equipment to the facilities. Further, the providers in CHPS compounds recounted that persons with physical disabilities that often develop secondary conditions such as sores needed care. However, participants said a lack of the necessary supplies especially in CHPS compounds impeded the provision services to such clients. A participant emphasized this point: *we sometimes run out of medical supplies like gauze and hand gloves to provide the necessary services to persons with severe physical disabilities that develop sores*.

#### Limited healthcare providers

A limited number of providers was another key barrier noted in some of the clinics and health centers. More specifically, limited specialists at the PHC level to manage common conditions experienced by persons with physical disabilities was a key barrier for providers in providing optimal care for these clients. A provider in a health center summed up this experience: *One day a client with disability came to me with muscle spasm but I was unable to provide treatment to her because I do not have the necessary training and also we don’t have any specialist at this level to address the needs of this client*. Further, some of the participants in these facilities recounted that their staff numbers could not match with clients’ needs. Some providers in health centers indicated that their staffs were inadequate and due to this, they could not devote much time for their clients with severe physical impairments with secondary complications. This situation is often exacerbated during local market days. Participants especially expressed frustrations about high workload during market days since that is the only time clients from nearby communities often get reliable means of transportation. Although this situation pertains to both persons with and without disabilities, providers were more concerned that it often leads to delays in providing quality care to persons with physical disabilities.

#### Financial constraints

A majority of the participants (*n* = 12) cited financial constraints as a common challenge faced by their clients with physical disabilities. The participants especially indicated that although the national health insurance scheme covers most of the services, some of the clients with physical disabilities could not purchase or annually renew the insurance coverage because of poverty. Some of the providers (*n* = 8) attributed this issue to societal discrimination that often excludes disabled people from the job market. Unsurprisingly, they reported that many of their clients with disabilities were unemployed and thus lacked financial resources to cover the cost of care. An excerpt from a participant emphasized: *most of the clients with disabilities are not working and so when they come for care at times I have to just consider them because they find it difficult to pay for services. They are like abandoned people in society*. They also mentioned that clients with insurance coverage sometimes find it difficult to afford the indirect cost of care (ie, transportation) to health facilities.

#### Inaccessible facilities and equipment

Most of participants (*n* = 13) cited inaccessible facilities and medical equipment as a major barrier in providing service to their clients with physical disabilities. Providers lamented how health facilities were designed without taking the needs of clients with assistive devices into consideration. A participant recounted: *the design of this building is nothing to write home about. The entrance is steep that clients in tricycles can’t propel them into the facility. It is a shame that the architects did not factor the needs of those with mobility devices into the design and that is really bad*. The participants further highlighted that when the clients with tricycles manage to access the facility, they encounter different layers of barriers such as inaccessible rooms and tables. Some participants (*n* = 7) reported that weighing scales could not accommodate the needs of those with severe physical impairments. These participants recounted that the lack of disability-friendly equipment often resulted in devoting substantial time to provide services to persons with physical disabilities, and this partly contributed to long queues in some facilities.

### Theme 2: Navigating the challenges

#### Improvising techniques and employing professional values

Most of the providers (*n* = 13) explained that the lack of ramps and disability-friendly equipment means that they had to improvise techniques like guessing their clients’ weight to provide adequate care. A participant summed up this issue: *when they can’t stand on the weighing scale, we usually guess their weight and provide the necessary prescription. Is so frustrating but what else can we do?* Providers further indicated that when they encounter challenges such as high workload on market days, they employ professional values (eg, making self-sacrifices) by placing the health needs of the patients above their personal needs. A few of the providers (*n* = 4) also recounted the importance of making self-sacrifices despite the lack of incentives and compensation packages. For instance, they recalled that dedicating more time to clients with physical disabilities could prevent further secondary complications like sores.

#### Referring clients

In cases of limited supply of services (eg, drugs and equipment) and providers (eg, rehabilitation specialists), some participants (*n* = 9) indicated that they refer their clients to higher facilities (eg, health centers). These participants, however, noted that referring clients often makes them feel guilty about their inability to provide services to their clients with severe disabilities. A participant noted: *one afternoon two caregivers assisted a client with disability over here for services. Unfortunately, I had to refer this client to the next level and that made me feel guilty when I consider the struggle of the client in using public transport, and the associated cost and the time involved by the two caregivers in accompanying him to the next health facility. What else can I do? Because I have no choice either than to refer him*. The participants further recounted how clients often doubt their competency level when they refer these clients. Meanwhile, participants noted that this issue can be squarely placed with the inability of policymakers to make rehabilitation specialists available at the PHC level to handle the specific healthcare needs of their clients with physical disabilities.

#### Providing financial assistance to clients

Some of the providers (*n* = 8) recounted how they financially assisted their clients by providing services on credit or giving cash to meet the direct and indirect costs of care. For instance, a few of the participants (*n* = 5) held the view that most of the clients with disabilities, they often see, could not afford their basic needs. Hence, when these clients visit health facilities for care, providers often attend to them and give them drugs on credit. Similarly, the participants also recounted that they financially assisted clients with disabilities to purchase drugs at local drugstores and also cover transportation cost back to their community. One participant explained why they are often obliged to provide such financial assistance: *some of these clients with disabilities can’t even afford food let alone medical expenses. There was a day a client came and told me how she struggled to get money to come here for care. Her story was so sad and I had to just give her money to buy drugs and transportation back home*.

### Theme 3: Positive experiences in providing healthcare

#### Feeling rewarded and appreciated in rural practice

Some of the participants (*n* = 7) indicated it is rewarding to serve clients with physical disabilities in rural communities. For instance, a participant explained how a client in tricycle inspired her to continue working with clients with disabilities. She explained: *Although I was not able to provide the needed services to a client with tricycle with muscle spasm, she was happy I dedicated sometime to listen to her problems and that inspires me to still stay in this community. It gives me the feeling that such clients need me here*. These participants further explained that rural practice enables them to reap rewards such as being considered as ‘experts’ in handling different conditions of disabled people because they are always the first contact for these clients in rural communities. Additionally, participants expressed their excitement about how rural residents with disabilities appreciate the services they often provide. Participants particularly shared that family members of clients with disabilities often demonstrate appreciation for their work by giving gifts of farm produce.

## Discussion

This study provides insights into the perspectives of providers in providing PHC services to clients with physical disabilities in rural Ghana. These perspectives include challenges providing care, navigating the challenges, and positive experiences providing care.

Overall, the results suggest that providers encountered multiple barriers that are in accordance with previous studies globally wherein inaccessible facilities and equipment, insurance constraints, and limited supply of services and providers were identified as the common barriers to quality healthcare delivery to clients with physical disabilities (Knox *et al*., 2014; Vergunst *et al*., 2015; Mitra *et al*., 2017; Smeltzer *et al*., 2018). Importantly, evidence has shown that the limitations of providing PHC services are systemic issues across Ghana, with clients with disabilities experiencing more access barriers than the general population as a result of the systemic problems (Dassah *et al*., 2018b). In particular, the limited availability of services and providers in meeting the specific health needs of clients with disabilities were recurrent barriers the participants highlighted in this study. Similar to existing evidence, the lack of services and providers especially rehabilitation specialists in rural areas is a global concern and negatively impacts disabled people in receiving care (Roots and Li, 2013; Hussain and Tait, 2015; Dassah *et al*., 2018a). Some of the participants in this study, however, recounted that they had to cope with these barriers by referring clients. Nevertheless, research has indicated that financial predicaments (ie, cost of care and transportation) and long distance to facilities coupled with unknown dangers often deter referral uptake for disabled people to health facilities (Bedford *et al*., 2013). This point was especially noted by the participants in this study and they often feel uncomfortable in referring their clients to service centers. In view of this, researchers have suggested that the provision of mobile vans/clinics could enhance disabled people’s access to healthcare services in rural Ghana (Ganle *et al*., 2016; Dassah *et al*., 2018b). Additionally, strategies may be targeted toward recruitment of rehabilitation professionals to augment the human resource level of providers to improve equitable access to healthcare (Roots and Li, 2013). However, in cases of professional shortage, the World Report on Disability recommends that training of community workers in LMICs could enhance the provision of basic rehabilitation interventions for disabled people at the PHC level (World Health Organization, 2011).

Another important barrier raised by participants was the inability of their clients with disabilities to afford healthcare. Thus, while Ghana’s health insurance was introduced to reduce financial barriers to access, inequities in access still exist between disabled people and the general population (Badu *et al*., 2015). This situation can be attributed to the cyclical nature of disability and poverty (Yeo and Moore, 2003; Banks *et al*., 2017). For instance, participants’ narratives point to systemic factors (eg, discrimination) that generally exclude clients with disabilities from the job market. This scenario often pushes disabled people into poverty, and because of this, they may not be able to purchase or renew their health insurance and afford out-of-pocket payment for services. This financial situation could exacerbate their disabling conditions as they find it difficult to access healthcare when needed. As a result, participants recounted providing personal financial assistance to cover clients direct and indirect cost of services. Nevertheless, evidence shows that this strategy is often unsustainable and insufficient (Hutchison *et al*., 2017), and could also paint disabled people as ‘objects of charity’. Considering this, policymakers need to fully commit to the implementation of Ghana’s Disability Act provision on free medical care and employment opportunities for disabled people. This can be achieved through intersectoral collaborations among stakeholders in the ministries of social protection, health, and employment. More specifically, collaboration between employment and social protection ministries could foster skill training and employment opportunities for disabled people to reduce poverty (Naami *et al*., 2012).

Taken together, Russell *et al*.’s (2013) conceptualization of access as a ‘fit’ between the characteristics of healthcare systems (supply) and client characteristics (demand) is analytically useful in explaining the access barriers. Thus, the barriers reported by the participants in this study illustrate how the complex interplay of supply and demand determines healthcare access. For instance, we noted that access barriers emanated from the inability of the healthcare system to match the specific healthcare needs of clients with physical disabilities. This pattern also reflects an earlier study which noted that a mismatch between supply and demand often results in barriers to healthcare access for persons with physical disabilities in rural Ghana (Dassah *et al*., 2018b).

A central theme in the analysis was how participants improvised techniques and employed professional values through self-sacrifices to navigate access barriers. The focus on employing professional values is consistent with research on providing care for clients with multiple chronic conditions (Loeb *et al*., 2016). Providers’ commitment to professional values, especially through self-sacrifices, is one of the core tenets of health professional bodies and agencies globally. For instance, the core tenets of professional values (eg, self-sacrifices) outlined by Ghana Health Service may include demonstrating high sense of dedication to duty and upholding the dignity and interest of clients without discrimination (Ghana Health Service, 2003). It is worth noting that providers’ adherence to professional values in the light of resource constraints may have negative consequences. For example, participants’ narratives of working long hours and relegating their personal needs to satisfy clients’ healthcare needs could contribute to provider burnout (Loeb *et al*., 2016). This burnout could give rise to both physical and psychosomatic problems (eg, anxiety, depression, and low tolerance of frustration) that could be transferred to clients (Suñer-Soler *et al*., 2014). This may be a possible factor that leads to clients casting providers in a negative light. Therefore, the availability of rehabilitation professionals at the PHC level is crucial in reducing burnout of existing staff.

Participants recounted positive experiences that enable them to provide high quality of care to clients with disabilities. This finding contrasts with research that described adversarial relationships between providers and disabled people in Ghana (Badu *et al*., 2016b). Participants in this study particularly highlighted the appreciating and rewarding experience for providing care to persons with physical disabilities. They also reported that they had a cordial working relationship with these clients. Providers however described systemic challenges (ie, lack of providers, drugs and medical equipment, and inaccessible building) that hamper the delivery of quality care to their clients with disabilities. These patterns mirror a recent study highlighting that while clinicians generally experienced positive encounters in caring for pregnant women with physical disabilities, systemic issues (ie, inaccessible facilities) tend to frustrate their efforts in providing quality care (Smeltzer *et al*., 2018). As such, providers’ frustrations with the systemic barriers compelled them to take some decisions (eg, referring clients and improvising techniques). However, such decisions could potentially attract negative remarks from clients. Despite these systemic issues, the positive encounters between providers and clients could serve as useful guide for providers in different LMICs to learn and promote client-centered services in rural communities.

The participants’ positive attributes also challenge a deficit discourse that dominates in rural health. The deficit discourse refers to the deficiencies of rural health when compared to urban health (Bourke *et al*., 2010; Malatzky and Bourke, 2016). Bourke *et al*. (2010) especially noted that rural health practitioners, policy makers, and researchers persistently emphasize this deficit discourse, which maintains and reinforces ‘problem-describing’. Consequently, the authors have commented on the need to focus on the strengths of rural health and promote it as ‘problem-solving’ (Bourke *et al*., 2010). Therefore, participants’ positive encounters with their clients with physical disabilities could potentially motivate them to work with disabled people in rural communities. This can also increase providers’ knowledge and skills in managing health issues encountered by disabled people in rural areas of resource-poor settings.

## Limitations

This study was conducted in few selected districts and municipal assemblies of rural Ghana. This makes it difficult to transfer the findings to other rural areas. Another limitation is that high workload and tight schedules of providers could not allow for more frequent or longer interviews. Although we determined that information power was reached, there is the possibility that more information would have emerged in a larger sample size. Notwithstanding these limitations, the results provide interesting perspectives that are congruent with similar emerging studies on providers’ perspectives especially around the challenges in providing healthcare to disabled people (Knox *et al*., 2014; Mitra *et al*., 2017; Maragh-Bass *et al*., 2018; Smeltzer *et al*., 2018). The study also offers insights about the development of policy interventions and clinical practices aimed at improving the quality of healthcare to persons with physical disabilities in rural Ghana.

## Conclusion

This study contributes to the nascent evidence that addresses the perspectives of providers in healthcare delivery to disabled people by incorporating the perspectives of those in rural Ghana. This study particularly supports existing evidence on positive provider–client with physical disabilities’ interactions and the challenges in healthcare provision to these clients (Smeltzer *et al*., 2018). This study is unique in that we expanded the existing evidence by explaining a strength-based approach in healthcare delivery to disabled people, thus counteracting the deficit discourse in rural health. The study also delved into strategies employed by providers in dealing with access barriers and noted that the strategies are likely unsustainable. We, therefore, recommended possible areas of policy, practice, and research that could ensure sustainable service delivery. Importantly, the findings reinforce the need for policymakers to consider the availability of rehabilitation professionals and services to address the specific healthcare needs of disabled people at the PHC level in Ghana. The findings also point to a need for further research on the perspectives of healthcare policymakers about how to navigate the systemic barriers encountered by providers in providing care to persons with physical disabilities in rural Ghana in particular, and other similar rural areas.
